# NF-κB System Is Chronically Activated and Promotes Glomerular Injury in Experimental Type 1 Diabetic Kidney Disease

**DOI:** 10.3389/fphys.2020.00084

**Published:** 2020-02-11

**Authors:** Orestes Foresto-Neto, Amanda Helen Albino, Simone Costa Alarcon Arias, Viviane Dias Faustino, Fernanda Florencia Fregnan Zambom, Marcos Antonio Cenedeze, Rosilene Motta Elias, Denise Maria Avancini Costa Malheiros, Niels Olsen Saraiva Camara, Clarice Kazue Fujihara, Roberto Zatz

**Affiliations:** ^1^Renal Division, Department of Clinical Medicine, Faculty of Medicine, University of São Paulo, São Paulo, Brazil; ^2^Nephrology Division, Department of Medicine, Universidade Federal de São Paulo, São Paulo, Brazil

**Keywords:** diabetic kidney disease, glomerulosclerosis, NF-κB, innate immunity, pyrrolidine dithiocarbamate

## Abstract

High glucose concentration can activate TLR4 and NF-κB, triggering the production of proinflammatory mediators. We investigated whether the NF-κB pathway is involved in the pathogenesis and progression of experimental diabetic kidney disease (DKD) in a model of long-term type 1 diabetes mellitus (DM). Adult male Munich-Wistar rats underwent DM by a single streptozotocin injection, and were kept moderately hyperglycemic by daily insulin injections. After 12 months, two subgroups – progressors and non-progressors – could be formed based on the degree of glomerulosclerosis. Only progressors exhibited renal TLR4, NF-κB and IL-6 activation. This scenario was already present in rats with short-term DM (2 months), at a time when no overt glomerulosclerosis can be detected. Chronic treatment with the NF-κB inhibitor, pyrrolidine dithiocarbamate (PDTC), prevented activation of renal TLR4, NF-κB or IL-6, without interfering with blood glucose. PDTC prevented the development of glomerular injury/inflammation and oxidative stress in DM rats. In addition, the NF-κB p65 component was detected in sclerotic glomeruli and inflamed interstitial areas in biopsy material from patients with type 1 DM. These observations indicate that the renal NF-κB pathway plays a key role in the development and progression of experimental DKD, and can become an important therapeutic target in the quest to prevent the progression of human DKD.

## Introduction

The pathogenesis of Diabetic Kidney Disease (DKD) remains elusive, despite the advancements obtained in the past few decades. Little therapeutic improvement has been introduced since the advent of ACEI and ARBs in the 1980s and 1990s. Since DKD remains a leading cause of chronic kidney disease (CKD), the development of novel therapeutic strategies is badly needed.

A number of recent observations from our group indicates that activation of innate immunity may exert a key role in the pathogenesis of non-diabetic CKD (Fujihara et al., 2007; Okabe et al., 2013; Faustino et al., 2018; Foresto-Neto et al., 2018; Ávila et al., 2019). In particular, the nuclear factor kappa B (NF-κB) cascade was shown to be set off in rats with 5/6^ths^ renal ablation or adenine overload, whereas NF-κB inhibition with pyrrolidine dithiocarbamate (PDTC) was shown to prevent the development of renal injury in these CKD models (Fujihara et al., 2007; Okabe et al., 2013). However, the role of NF-κB activation in DKD has not been unequivocally demonstrated. A previous study of STZ-diabetic rats demonstrated activation of the renal NF-κB system, and showed that treatment with PDTC reverted renal inflammation at an early phase (Lee et al., 2004). However, the possibility that PDTC may prevent the long-term development of glomerulosclerosis was not verified in that study.

In the present study, we sought to investigate whether NF-κB activation is associated not only with the development of early and late glomerular inflammation, but also with the long-term development of clear-cut glomerular sclerotic lesions in STZ-diabetic rats. To accomplish this goal, we investigated whether the presence of NF-κB would predict the development of DKD 12 months after induction of DM. In a second protocol, we treated DM rats with PDTC for a year, to investigate whether long-term NF-κB inhibition can become a strategy to prevent or detain the development of DKD.

## Materials and Methods

### Induction of Diabetes Mellitus

Adult male Munich-Wistar rats obtained from a local facility, with initial body weights (BW) ranging from 230 to 250 g, were used in this study. Rats received a single i.v. injection of STZ (Sigma Chemical, St. Louis, MO, United States), 65 mg/Kg. DM was confirmed 2 days later by reflectometric measurement of blood glucose concentration (BG) in tail blood samples. All diabetic rats received evening injections of NPH insulin (Novo Nordisk, Kalundborg, Denmark), individually adjusted to maintain BG between 350 and 450 mg/dL, in order to model the usual clinical scenario, in which control of blood glucose levels is imperfect, rather than non-existent. Daily insulin doses ranged from 1 to 4 units/rat. Non-diabetic rats matched for initial age and BW, and given no pharmacological treatment, were used as controls (C). All rats had free access to tap water and standard rodent chow containing 0.5% Na and 22% protein (Nuvital Labs, Curitiba, Brazil), and were kept at 22 ± 1°C and 60 ± 5% relative air humidity under an artificial 12:12-h light–dark cycle. All experimental procedures were specifically approved by the local Research Ethics Committee (CAPPesq, process no. 034/15) and followed strictly international standards for manipulation and care of laboratory animals.

### Experimental Groups

Thirty-two DM rats and 29 matched non-diabetic controls (C) were followed for 12 months after STZ injection, with biweekly determination of BW and BG. At the end of the 12-month period of study, two subgroups of DM rats were formed: DKD−, the 10 DM rats with the least percentage of sclerotic glomeruli (%GS); and DKD+, the 10 DM rats with the highest %GS. Twelve representative C rats were used for comparison. An additional cohort of 7 DM and 5 C rats was kept under conditions identical to those described above, and followed for 2 months after STZ injection.

### Effect of Inhibition of the NF-κB System

In a separate cohort, 11 STZ-DM rats received the NF-κB inhibitor PDTC (Sigma Chemical, St. Louis, MO, United States), dissolved in the drinking water, 60 mg/Kg/day (Group DM+PDTC), and 16 STZ-DM rats receiving vehicle (pure water) only were followed during 12 months. Both DM groups received daily insulin injections and underwent determination of BW and BG.

### Histologic Techniques and Histomorphometric Analysis

At the end of each period of observation, rats were anesthetized with ketamine (50 mg/kg im) and xylazine (10 mg/kg im). The right kidney was retrogradely perfused *in situ* through the abdominal aorta with saline to remove blood from renal vessels, excised and rapidly frozen at −80°C for protein assessment and/or isolation of nuclei. The left kidney was perfused *in situ* with saline and after with Dubosq-Brazil solution for fixation. Two midcoronal slices of the right kidney were postfixed in buffered 10% formaldehyde solution, and embedded in paraffin. Four-μm-thick kidney sections were used for histomorphometric and immunohistochemical analyses.

For the evaluation of the glomerular damage, 4-μm-thick kidney sections were stained by the periodic acid-Schiff reaction (PAS). The extent of glomerular injury was estimated by determining the percentage of glomeruli with sclerotic lesions, as described previously (Teles et al., 2009).

### Immunohistochemical Analysis

Four-μm-thick renal sections were mounted on 2% silane coated glass slides. The following primary antibodies were used: polyclonal rabbit anti-zonula occludens 1 (ZO-1) (Invitrogen, Waltham, MA, United States); monoclonal mouse anti-rat ED-1 (Serotec, Oxford, United Kingdom) for macrophages; polyclonal anti-NLRP3 (Novus Biologicals, Littleton, CO, United States); monoclonal rabbit anti-total p65 (Cell Signaling, Danvers, MA, United States) for the NF-κB system. Details of the technique for immunohistochemistry are given elsewhere (Arias et al., 2013; Foresto-Neto et al., 2018). The glomerular density of ED-1-positive cells was evaluated in a blinded manner at ×400 magnification. The glomerular area staining positively for ZO-1 or NLRP3 was estimated by a point-counting technique, under ×400 magnification. For each section, fifty microscopic fields in cortical area (corresponding to a total area of 1.6 mm^2^) were examined.

Human renal tissue was obtained from archived paraffin-embedded biopsy material kept at the Hospital das Clínicas, a tertiary academic health center affiliated with the University of São Paulo. Five renal sections were obtained from Type 1 DM patients with clinical suspicion of an associated primary glomerulopathy, who received a diagnosis of DKD alone after careful histologic analysis by a qualified pathologist. Normal renal tissue was obtained from a patient who died from a stabbing injury. Four-μm-thick renal sections were pretreated with 30% hydrogen peroxide in methanol. Non-specific binding was blocked with the Protein Block Serum-Free reagent (Dako, Glostrup, Denmark). The monoclonal anti-p65 primary antibody (Cell Signaling, Danvers, MA, United States) was diluted at 1:800 in 1% BSA. After rinsing with Tris-buffered saline, sections were incubated with HRP-labeled polymer conjugated with secondary antibodies (Dako, Glostrup, Denmark), then with DAB substrate-chromogen solution (Dako, Glostrup, Denmark) for development. Use of the human material was approved by the local ethical review board (no. 45163715.4.0000.0068).

### Western Blot Assays

The extraction of renal proteins and the isolation of the nuclei from renal cortical tissue were performed as described elsewhere (Foresto-Neto et al., 2018). Briefly, 100 μg of total proteins were mixed with Laemmli loading buffer and were denatured (except in the case of the nuclear moiety) at 96°C for 5 min. Protein separation was performed by sodium dodecyl sulphate polyacrylamide gel electrophoresis, with subsequent transfer to a nitrocellulose membrane, which was incubated with 5% non-fat milk or 5% BSA in TBS for 2 h at room temperature to block non-specific binding. The membranes were then incubated overnight at 4°C with the following primary antibodies: polyclonal anti-TLR4, 1:250 (Santa Cruz Biotechnology, Santa Cruz, CA, United States); monoclonal anti-phosphorylated NF-κB p65 component, 1:100 (Cell Signaling, Danvers, MA, United States); polyclonal anti-histone H2B, 1:1500 (Abcam, Cambridge, United Kingdom); monoclonal anti-IL-6, 1:1000 (Abcam, Cambridge, United Kingdom); monoclonal anti-HMGB1, 1:10000 (Abcam, Cambridge, United Kingdom); monoclonal anti-heme oxygenase 1 (HO-1), 1:500 (Abcam, Cambridge, United Kingdom); polyclonal anti-superoxide dismutase 2 (SOD2), 1:10000 (Cayman, Ann Arbor, MI, United States); monoclonal anti-β-actin, 1:5000 (Sigma-Aldrich, St. Louis, MO, United States). After rinsing with TBS Tween 20 buffer, the membranes were incubated with appropriate secondary antibodies labeled with HRP. A chemiluminescence kit (Thermo Fisher Scientific, Rockford, IL, United States) was used to detect immunolabeled bands, which were further analyzed by densitometry with a gel documentation system and the Uvisoft-UvibandMax software (Uvitec Cambridge, Cambridge, United Kingdom). The full-length blots are shown in Supplementary Information.

### ELISA Assay

The serum concentration of monocyte chemoattractant protein 1 (MCP-1) was measured using a commercial kit (R&D Systems, Minneapolis, MN, United States), in accordance with the manufacturer’s instructions.

### Statistical Analysis

The results are expressed as means ± SEM. One-way ANOVA was used to assess statistical differences among groups (with Newman–Keuls post-test) (Wallenstein et al., 1980), and the Student’s unpaired *t*-test was used when the comparison involved two groups. The Pearson’s correlation coefficient was calculated to ascertain the existence of linear correlation between the expression of p65 and the %GS. *p* < 0.05 was considered significant. All calculations were performed using GraphPad Prism 4.0 software.

## Results

### NF-κB Signaling Is Overactivated Only in Those DM Animals Which Developed Glomerular Injury After 12 Months

**FIGURE 1:**
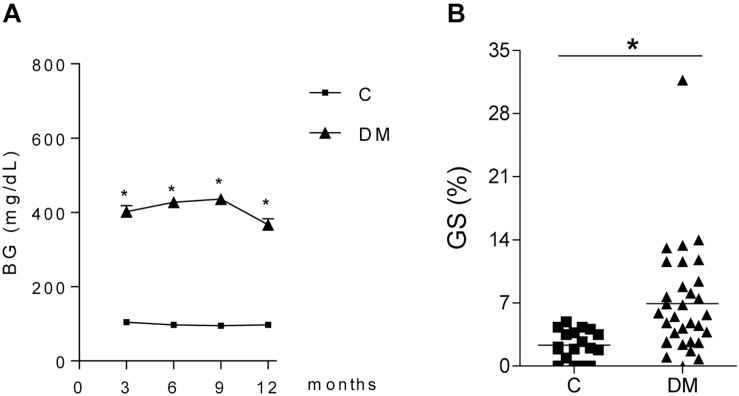
Time course of **(A)** blood glucose concentration (BG, mg/dL) in diabetic (DM, *n* = 32) and control (C, *n* = 29) rats. **(B)** shows the frequency of glomeruli with sclerotic lesions (GS,%) in C and DM at 12 months. Results expressed as means ± SE. * *p* < 0.05 vs. C.

**FIGURE 2:**
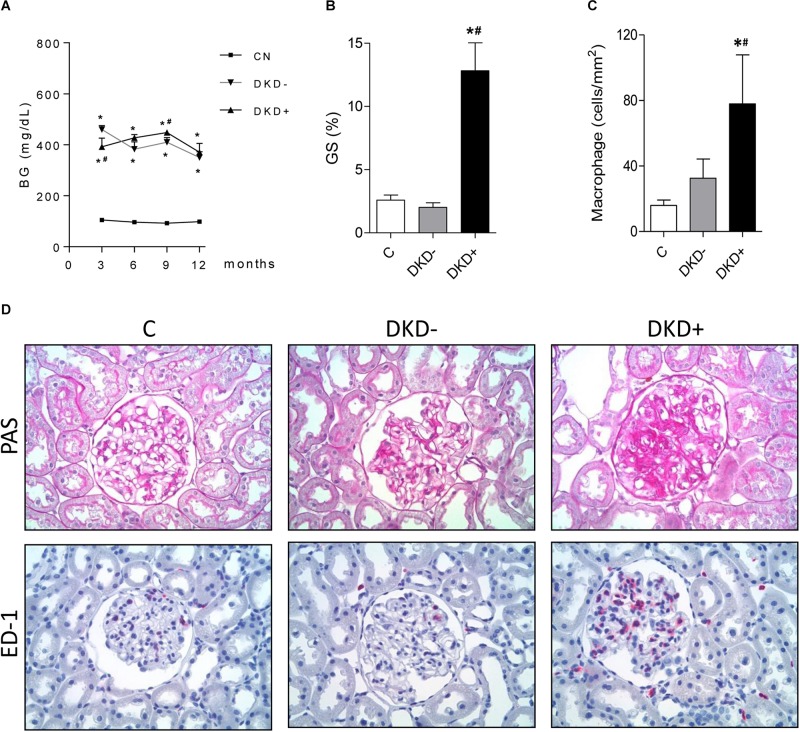
Twelve months after STZ injection, the 10 DM rats with the highest values for percent glomerulosclerosis (%GS) were used to create the DKD+ group, whereas the 10 DM rats with the lowest %GS values constituted the DKD– group. Twelve non-diabetic age-matched rats were taken as controls (C). The time course of blood glucose concentration (BG, mg/dL) is shown in panel **(A)**, whereas the %GS and glomerular macrophage density (ED-1, cells/mm^2^) at 12 months of DM appear at panels **(B,C)**, respectively. Representative microphotographs of renal tissue stained with PAS (for %GS) or immunohistochemistry (for macrophage-specific ED-1 antigen) are shown in panel **(D)** (x400). Results expressed as means ± SE. * *p* < 0.05 vs. C, ^#^*p* < 0.05 vs. DKD–.

**FIGURE 3:**
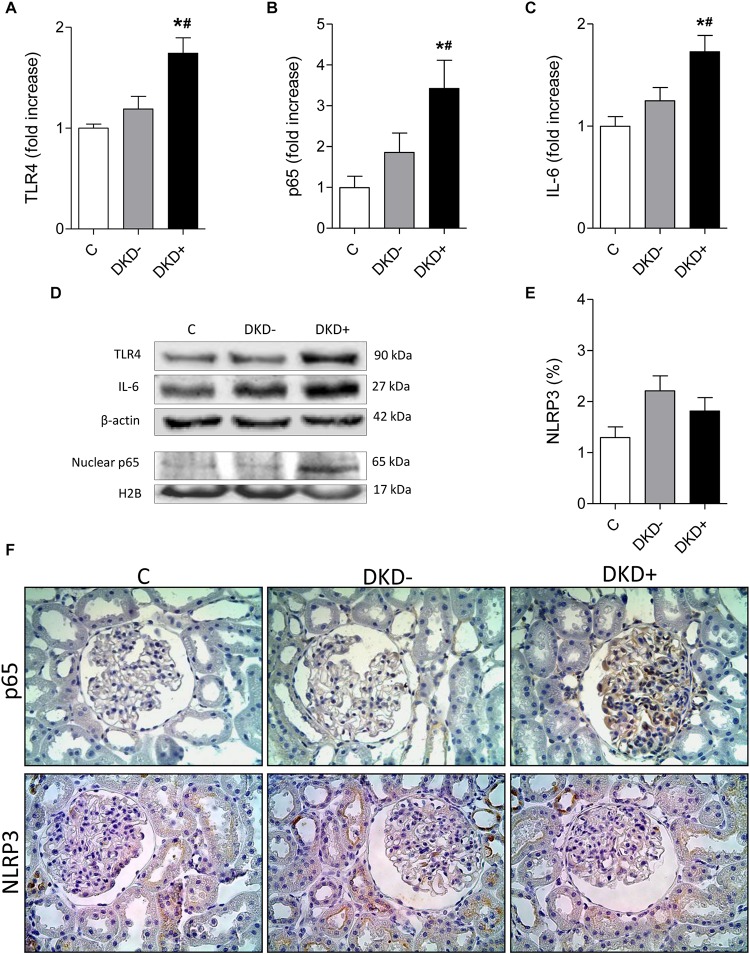
Diabetic rats with (DKD+ group, *n* = 10) or without (DKD– group, *n* = 10) significant glomerular injury were analyzed 12 months after streptozotocin injection. Twelve non-diabetic age-matched rats were taken as controls (C group). The renal cortical contents of **(A)** Toll-like receptor 4 (TLR4), **(B)** nuclear fraction of phosphorylated NF-κB component (p65), and **(C)** interleukin 6 (IL-6), were quantified using **(D)** western blot analysis. Panel **(E)** shows the percent glomerular area staining for NLRP3 (%) quantified by immunohistochemistry. **(F)** Representative microphotographs show the presence of p65-positive or NLRP3-positive immunostaining (brown) in glomeruli (×400). No difference in NLRP3 positivity was observed between groups DKD– (non-progressors) and DKD+ (progressors). Results expressed as means ± SE. * *p* < 0.05 vs. C, ^#^*p* < 0.05 vs. DKD–.

Thirty-two STZ-DM rats were kept moderately hyperglycemic for 12 months (Figure 1A). After 12 months, a significant increase was observed in the mean %GS (Figure 1B). Since only about 40% of DM rats developed glomerular injury at this time, we analyzed two subgroups, composed by rats exhibiting the lowest 10 (Group DKD−) and the highest 10 (Group DKD+) %GS values, to investigate possible differences in the NF-κB system. BG levels (Figure 2A) and insulin doses needed (Supplementary Figure 1) were similar in both DM groups along the study. As expected, %GS and macrophage infiltration were significantly increased in Group DKD+ compared with DKD− (Figures 2B–D). These changes were associated with an increased renal content of TLR4, nuclear p65 and IL-6, whereas the renal content of NLRP3 was not changed (Figures 3A–F). No activation of TLR4, NF-κB, IL-6 or NLRP3 was detected in DKD− rats (Figures 3A–F). Immunohistochemical analysis showed that, in DKD+ rats, p65 located in glomeruli and, to a much lesser degree, in the interstitial area (Figure 3F). When all 32 1-year DM rats were considered, a significant linear correlation was observed between the renal expression of p65 in the nuclear fraction and the %GS (*R*^2^ = 0,338; *p* < 0.05) at this time point (Supplementary Figure 2). As a whole, these findings indicate that the renal NF-κB pathway is activated only in those DM rats that developed glomerular injury.

### TLR4/NF-κB Activation Preceded Glomerular Injury in DM Rats

**FIGURE 4:**
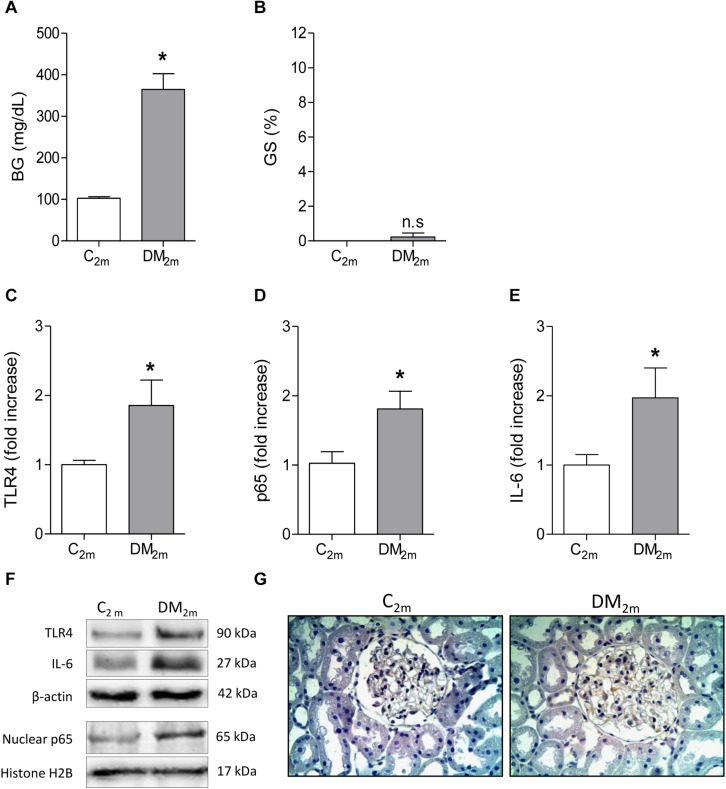
Blood glucose concentration (BG, mg/dL) and frequency of glomeruli with sclerotic lesions (GS,%), observed in Control (C_2__m_ group, *n* = 5) and DM (DM_2__m_ group, *n* = 7) rats 2 months after STZ injection, are shown in panels **(A,B),** respectively. The renal cortical contents of Toll-like receptor 4 (TLR4) **(C)**, nuclear fraction of phosphorylated p65 **(D)** and interleukin-6 (IL-6) **(E)** were quantified using Western blot analysis **(F)**. In panel **(G),** representative microphotographs show the presence of p65-positive immunostaining (brown) in glomeruli (×400). Results expressed as means ± SE. * *p* < 0.05 vs. C_2__m_.

To investigate whether renal TLR4 and NF-κB activation antedates overt glomerulosclerosis, seven rats received STZ and were kept moderately hyperglycemic for 2 months (Figure 4A) as described before. Although significant glomerulosclerosis was not yet present (Figure 4B), DM rats already exhibited augmented renal content of TLR4 (Figures 4C,F), higher nuclear translocation of the p65 subunit (Figures 4D,F), while p65 was detected in the glomerular area (Figure 4G). Accordingly, the renal abundance of IL-6 was increased (Figures 4E,F).

### Pyrrolidine Dithiocarbamate Inhibited the NF-κB Pathway and Prevented DKD

**FIGURE 5:**
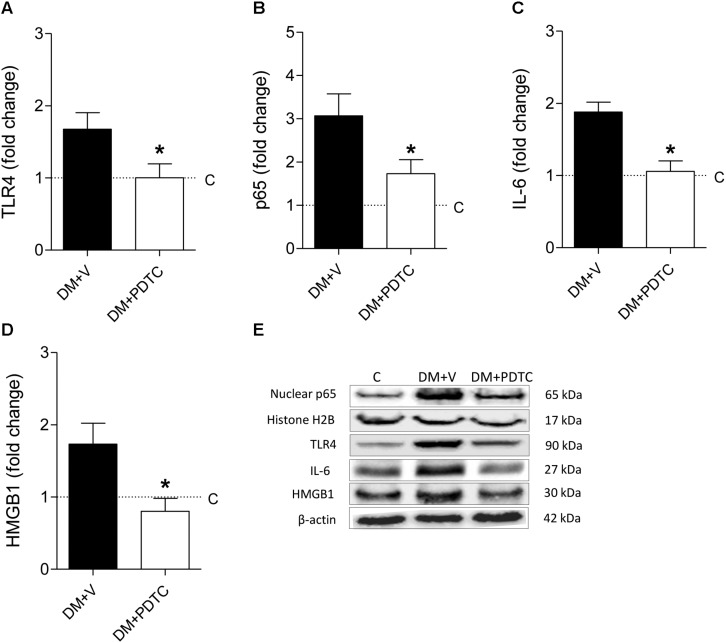
DM rats treated with the NF-κB inhibitor pyrrolidine dithiocarbamate (PDTC) (DM+PDTC group, *n* = 11) or vehicle (DM+V, *n* = 16), were followed for 12 months. At the end of this period, the renal cortical content of **(A)** Toll-like receptor 4 (TLR4), **(B)** nuclear fraction of phosphorylated p65, **(C)** interleukin 6 (IL-6), and **(D)** high mobility group box protein-1 (HMGB1) were quantified using **(E)** Western blot analysis. Values obtained from groups DM+V and DM+PDTC were factored by age-matched controls rats (C, horizontal dotted lines). Results expressed as means ± SE. * *p* < 0.05 vs. DM+V.

**FIGURE 6:**
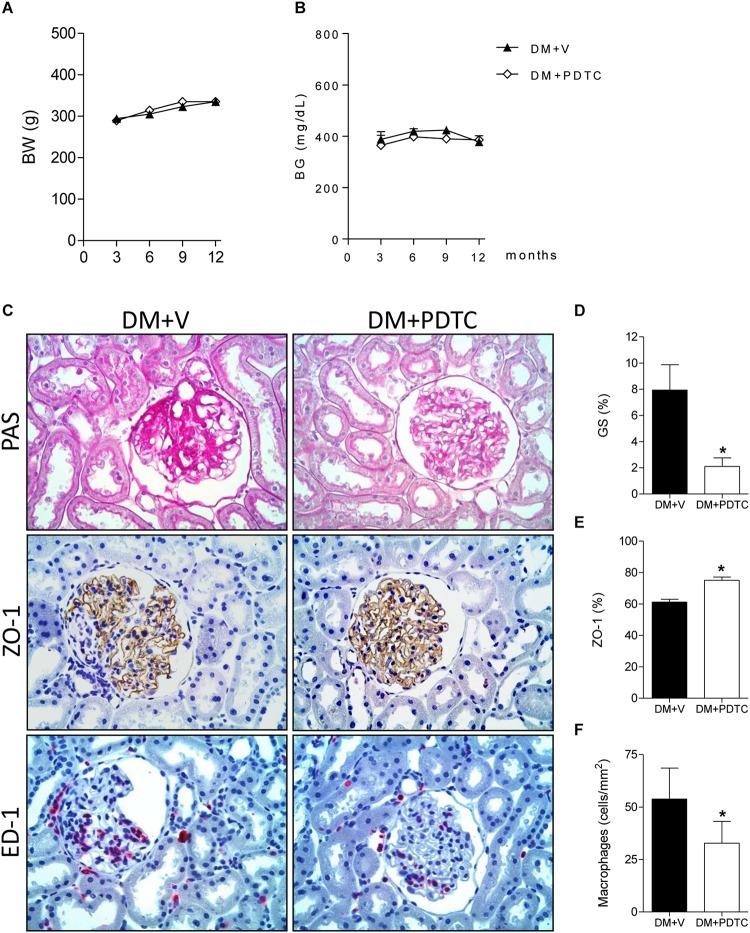
Time course of **(A)** body weight (BW, g) and **(B)** blood glucose concentration (BG, mg/dL) in rats treated with the NF-κB inhibitor pyrrolidine dithiocarbamate (Group DM+PDTC, *n* = 11) or vehicle (Group DM+V, *n* = 16) for 12 months. In panel **(C)**, illustrative microphotographs (×400) of glomerulosclerosis in PAS-stained renal tissue and detection, by immunostaining, of zonula occludens-1 (ZO-1) (brown), and macrophage (ED-1) (red) in renal tissue at 12 months of DM. Quantification of **(D)** frequency of glomeruli with sclerotic lesions (GS,%), **(E)** percent area of ZO-1 and **(F)** macrophage density (ED-1, cells/mm^2^) in the glomerular area at this time. Results expressed as means ± SE. * *p* < 0.05 vs. DM+V.

**FIGURE 7:**
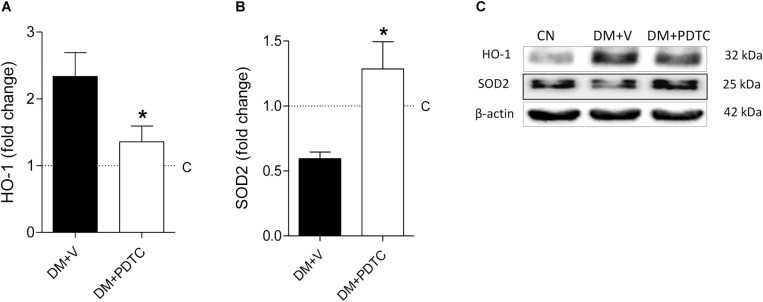
Renal cortical abundance of **(A)** heme oxygenase-1 (HO-1) and **(B)** superoxide dismutase 2 (SOD2), quantified by Western blot analysis **(C)** in rats treated with the NF-κB inhibitor pyrrolidine dithiocarbamate (Group DM+PDTC, *n* = 11) or vehicle (Group DM+V, *n* = 16) at 12 months of DM. The values obtained from the groups DM+V and DM+PDTC were factored by age-matched controls rats (C, horizontal dotted lines). Results expressed as means ± SE. * *p* < 0.05 vs. DM+V.

To directly assess whether the NF-κB system plays a role in the pathogenesis of the DKD, we treated DM rats with the NF-κB inhibitor, PDTC, for 12 months. PDTC efficiently reduced the renal content of TLR4, the p65 nuclear translocation and the increase of renal IL-6 and HMGB1 (Figures 5A–E), while exerting no effect on BW or BG (Figures 6A,B). Along with the NF-κB inhibition, PDTC effectively ameliorated the development of GS, the loss of ZO-1 and the glomerular infiltration by macrophages (Figures 6C–F). Of note, PDTC did not influence circulating MCP-1 levels (1088 ± 40 pg/mL vs. 1184 ± 73 pg/mL in DM+V, *p* > 0.05). Moreover, PDTC diminished the abundance of HO-1 and increased the content of SOD2 in renal tissue, indicating that oxidative stress was reduced by treatment (Figures 7A–C).

### NF-κB Was Detected in Human Type 1 DKD as Well

**FIGURE 8:**
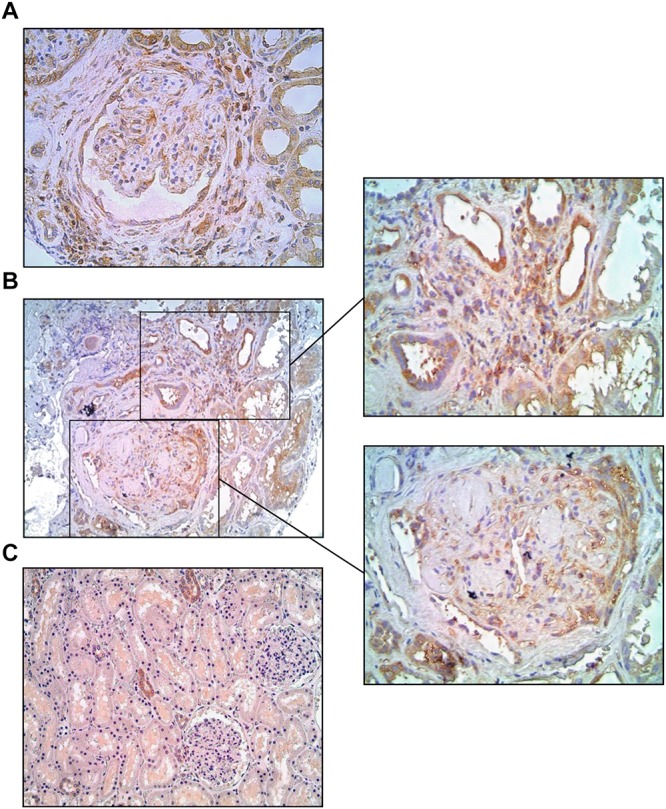
Microphotographs from Type 1 diabetic patients with advanced diabetic kidney disease, immunostained for p65 (brown). The p65 protein was detected in a relatively preserved glomerulus **(A)** and around Kimmelstiel-Wilson nodules in a more severely affected tuft **(B)**, as well as in inflamed interstitial areas **(A,B)**, whereas in normal renal tissue **(C)** only scattered tubular profiles were stained. Magnification: x200 **(B,C)** and x400 **(A** and insets).

To investigate whether NF-κB is also activated in the kidneys of Type 1 diabetic patients with DKD, as previously shown for Type 2 DM, we analyzed human renal biopsies with documented disease. Immunohistochemical analysis of renal tissue revealed positive staining for p65 in a relatively preserved glomerulus (Figure 8A) and around Kimmelstiel-Wilson nodules in a more severely affected tuft (Figure 8B), as well as in inflamed interstitial areas (Figures 8A,B). In normal renal tissue, only a few tubular profiles were stained (Figure 8C).

## Discussion

As shown previously (Zatz et al., 1986; Fujihara et al., 1992; Teles et al., 2009), rats kept moderately hyperglycemic exhibited widespread glomerular injury after 1 year of DM. Consistent with previous findings of this laboratory (Teles et al., 2009), no interstitial injury was seen in diabetic rats, supporting the view that, at least in the STZ model with daily insulin injection, and up to 1 year after DM induction, DKD is essentially confined to the glomeruli. Despite the significant differences between C and DM, a large dispersion of values was observed, to the point that two subgroups – progressors and non-progressors – could be formed based on the frequency of glomerular sclerotic lesions. This scenario is reminiscent of that observed in diabetic patients, only 20–40% of which develop DKD in the long run (Molitch et al., 2004; Gross et al., 2005).

Despite similar levels of hyperglycemia and similar daily insulin doses, only in progressors was activation of the NF-κB pathway observed, as indicated by the overexpression of TLR4, nuclear p65 and IL-6. It is noteworthy that only in the glomeruli was p65 detected by immunohistochemistry, reinforcing the notion that inflammation was restricted to the glomerular compartment. Consistent with this finding, these abnormalities were paralleled by intense glomerular macrophage infiltration, underlining the inflammatory nature of the glomerulopathy. Of note, the abundance of NLRP3 was not increased in progressors, indicating that, unlike the NF- κB system, this pathway of innate immunity was not activated in this subgroup. Accordingly, the extent of GS exhibited significant linear correlation with the nuclear phosphorylated p65 expression.

Previous observations from our group and elsewhere (Rangan et al., 2009) showed that the renal NF-κB system is activated in CKD caused by renal ablation (Fujihara et al., 2007), adenine overload (Okabe et al., 2013) and adriamycin nephropathy (Faustino et al., 2018). Accordingly, NF-κB inhibition with PDTC attenuated kidney injury and inflammation in two of these experimental models (Fujihara et al., 2007; Okabe et al., 2013).

NF-κB activation likely participates in the pathogenesis of DKD as well. In a previous study on STZ-diabetes (Lee et al., 2004), Lee and coworkers showed modest activation of the renal NF-κB system as early as 1 month after induction of DM. This abnormality was paralleled by intense glomerular inflammation, which was abrogated by treatment with PDTC. Glomerular inflammation, as well as moderate glomerulosclerosis, were also seen at 8 months of DM, although no attempt at preventing or reversing these abnormalities with PDTC was made in that study. Likewise, activation of renal TLR4 and NF-κB in the present study was demonstrated not only at 1 year of DM, but also as early as 2 months after STZ injection, at a time when established glomerular sclerotic lesions were absent. Together, these findings suggest that, in the STZ model, NF-κB activation is an early event that antecedes the development of glomerular inflammation and may participate in the pathogenesis of glomerulosclerosis. It is conceivable that rats exhibiting augmented p65 nuclear translocation at this time are those destined to develop DKD, although the available data cannot provide direct support for this concept.

The specific mechanisms leading to NF-κB activation in DM are unclear at this time. High ambient glucose was shown to induce TLR4 and NF-κB activation and upregulation of proinflammatory cytokines (Lin et al., 2012). NF-κB activation can also result from ligation of advanced glycation end products to specific receptors (RAGEs) (Doi et al., 1992; Yan et al., 1994; Wendt et al., 2003). In podocytes exposed to elevated glucose concentrations, the expression of TLR4 and MCP-1 was increased through a NF-κB-dependent pathway (Wei et al., 2015). Moreover, glomerular capillary hypertension and the resulting mechanical stretching of the glomerular cells, a well-known pathogenic factor (Utimura et al., 2003), can be potentiated by concomitant exposure to a high-glucose milieu, promoting inflammation via NF-κB activation (Gruden et al., 2005).

The present study is the first to show the beneficial effects of long-term inhibition of the renal NF-κB system in experimental diabetes, with consequent reduction of the production of IL-6, one of its main targets. Interestingly, PDTC also prevented the increase in the renal expression of TLR4 observed at this late time point. The mechanism for this effect is not immediately apparent. However, it must be noted that the expression of HMGB1, also increased in the diabetic group, was equally prevented by PDTC treatment. HMGB1 is a ligand for TLR4 and, at the same time, a product of NF-κB activation (Lin et al., 2012; Wang et al., 2016). Thus, increased HMGB1 production can result in the establishment of a positive feedback loop, which may contribute to extend and perpetuate a state of inflammation (van Beijnum et al., 2008).

PDTC treatment prevented the development of glomerulosclerosis, the loss of podocyte integrity (estimated by the expression of ZO-1) and the glomerular infiltration by macrophages observed in untreated rats after 12 months of DM. This beneficial effect cannot be explained by amelioration of hyperglycemia or systemic inflammation, as blood glucose levels and serum MCP-1 were not changed by treatment, but is closely associated to the efficient renal NF-κB inhibition provided by the drug. A possible additional mechanism for the salutary effect of PDTC is alleviation of oxidative stress, suggested by the observed normalization of the renal contents of HO-1 and SOD2 (Faustino et al., 2018; Foresto-Neto et al., 2018). This antioxidative action of PDTC was demonstrated previously in other models of CKD (Okabe et al., 2013; Yang et al., 2016). Since NF-κB activation can increase the production of reactive oxygen species (Anrather et al., 2006), which in turn can activate the NF-κB system, a positive feedback loop, analogous to that postulated for HMGB1 (van Beijnum et al., 2008), can arise (Morgan and Liu, 2011), contributing to the progressive nature of the glomerulopathy. Interruption of these vicious cycles is a plausible explanation for the prevention of DKD observed in these rats.

The relevance of NF-κB activation to the pathogenesis of human DKD has been addressed in a recent study, in which the p65 component was detected in sclerotic glomeruli from patients with Type 2 DM (Verzola et al., 2014). In the present study, we showed that a similar process takes place in patients with Type 1 DM. In both studies, the p65 molecule was detected not only in glomeruli, but also at inflamed areas of the renal interstitium. Since in this and in previous studies of experimental DM inflammation and NF-κB activation were shown to be confined to the glomeruli (Utimura et al., 2003), the finding of p65 at the interstitium of these patients suggests that NF-κB activation starts in glomeruli and then propagates to the interstitial area at more advanced phases. At any rate, these findings suggest that NF-κB activation plays an important role in the pathogenesis of both human and experimental DKD.

The main strength of this study is the finding that, in a large number of STZ diabetic rats followed for 1 year, development of advanced glomerular sclerotic lesions was closely associated with activation of the NF-κB pathway, and was prevented by inhibition of this system, raising the possibility that such a strategy may be useful against human DKD. In addition, this study showed that NF-κB activation may be a selective process, since activation of the NLRP3 was not detected. The main limitation of this study is that it did not test the possibility that NF-κB inhibition may ameliorate the progression of established kidney disease, although it should be noted that this would be a difficult task considering the high mortality observed in aging rats with DKD. Other limitation of our study is that we did not determine the HbA1C levels in progressor and non-progressor diabetic rats. However, given that blood glucose and the insulin doses needed to keep them at moderate levels were consistently similar between these groups along 1 year, it is unlikely that substantial differences existed regarding HbA1C levels.

In summary, our observations corroborate the notion that the renal NF-κB pathway is activated in glomeruli of STZ-DM rats developing DKD, a process that starts prior to the development of these lesions, and indicates that this is a selective process, since the NLRP3 inflammasome was not activated. NF-κB inhibition with PDTC prevented renal oxidative stress, glomerular inflammation and injury in these rats. Similar evidence of NF-κB activation was obtained in renal biopsy material from patients with DKD in both glomerular and interstitial areas. The NF-κB system can become an important therapeutic target in the quest to prevent the progression of human DKD.

## Data Availability Statement

The raw data supporting the conclusions of this article will be made available by the authors, without undue reservation, to any qualified researcher.

## Ethics Statement

This study was carried out in accordance with the recommendations of the local ethical review board, Comitê de Ética em Pesquisa do Hospital das Clínicas da Faculdade de Medicina da USP, with written informed consent from all subjects. The protocol was approved by the Comitê de Ética em Pesquisa do Hospital das Clínicas da Faculdade de Medicina da USP (no. 45163715.4.0000.0068). All experimental procedures were specifically approved by the local Research Ethics Committee (CAPPesq, process no. 034/15) and followed strictly international standards for manipulation and care of laboratory animals.

## Author Contributions

OF-N, AA, SA, VF, FZ, MC, RE, DM, CF, and RZ conceived and carried out the experiments and analyzed the data. RZ, CF, and NC conceived the research project. All authors were involved in writing the manuscript and had final approval of the submitted and published versions.

## Conflict of Interest

The authors declare that the research was conducted in the absence of any commercial or financial relationships that could be construed as a potential conflict of interest.
