# Supplementary material for: NF-κB System Is Chronically Activated and Promotes Glomerular Injury in Experimental Type 1 Diabetic Kidney Disease
Source: Front Physiol. 2020 Feb 11;11:84. doi: 10.3389/fphys.2020.00084 (PMC7026681; doi:10.3389/fphys.2020.00084)
Supplement: Supplementary file 1 [file Image_1.PDF]

NF-κB system is chronically activated and promotes glomerular injury in experimental type 1 diabetic kidney disease

Orestes Foresto-Neto [1]*, Amanda Helen Albino [1], Simone Costa Alarcon Arias [1], Viviane Dias Faustino [1], Fernanda Florencia Fregnan Zambom [1], Marcos Antonio Cenedeze [2], Rosilene Motta Elias [1], Denise Maria Avancini Costa Malheiros [1], Niels Olsen Saraiva Camara [1,2], Clarice Kazue Fujihara [1], Roberto Zatz [1]

1 Renal Division, Department of Clinical Medicine, Faculty of Medicine, University of São Paulo, São Paulo, Brazil

2 Nephrology Division, Department of Medicine, Universidade Federal de São Paulo, São Paulo, Brazil

Short running title: NF-κB in Long-term Diabetic Nephropathy

Correspondence and reprint request author:

Orestes Foresto-Neto
Faculdade de Medicina da Universidade de São Paulo
Avenida Dr. Arnaldo, 455
01246903 São Paulo-SP, Brazil
Tel.: +55-11-30618355
Email: forestoneto@usp.br

Supplementary Information

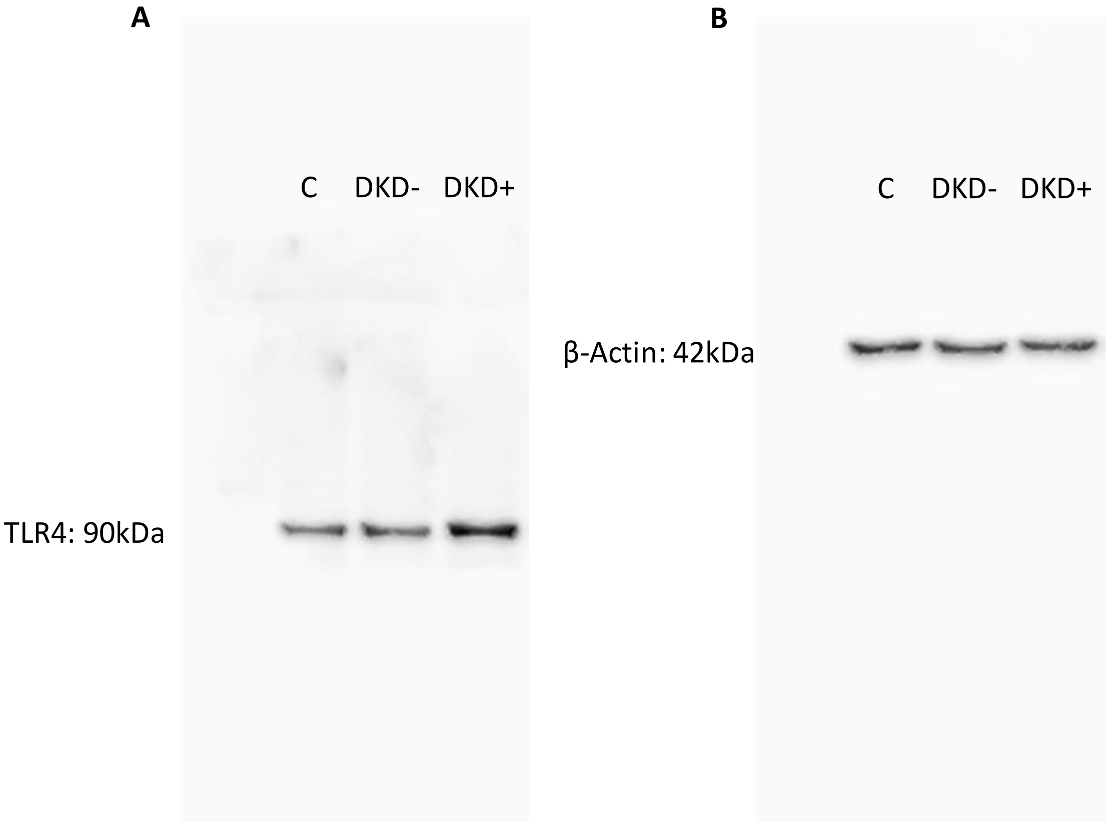

Full-length TLR4 (**A**) and constitutive β-Actin (**B**) representative Western Blots of groups C, DKD- and DKD+.

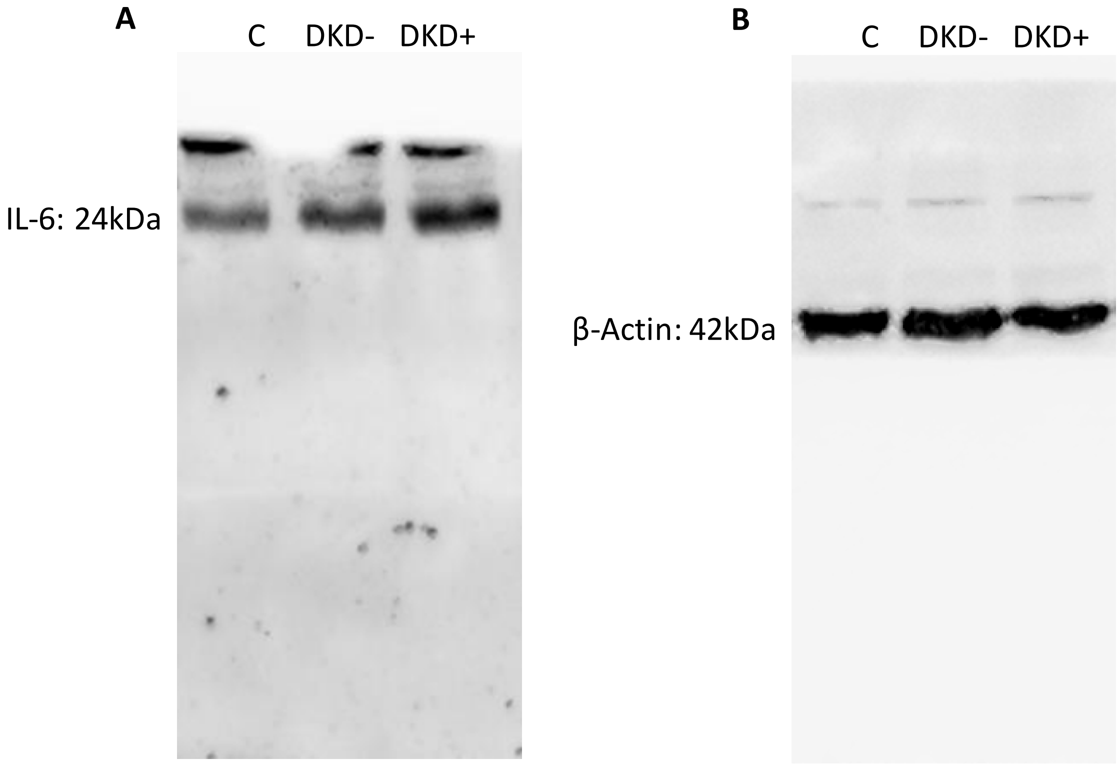

Full-length IL-6 (**A**) and constitutive β-Actin (**B**) representative Western Blots of groups C, DKD- and DKD+.

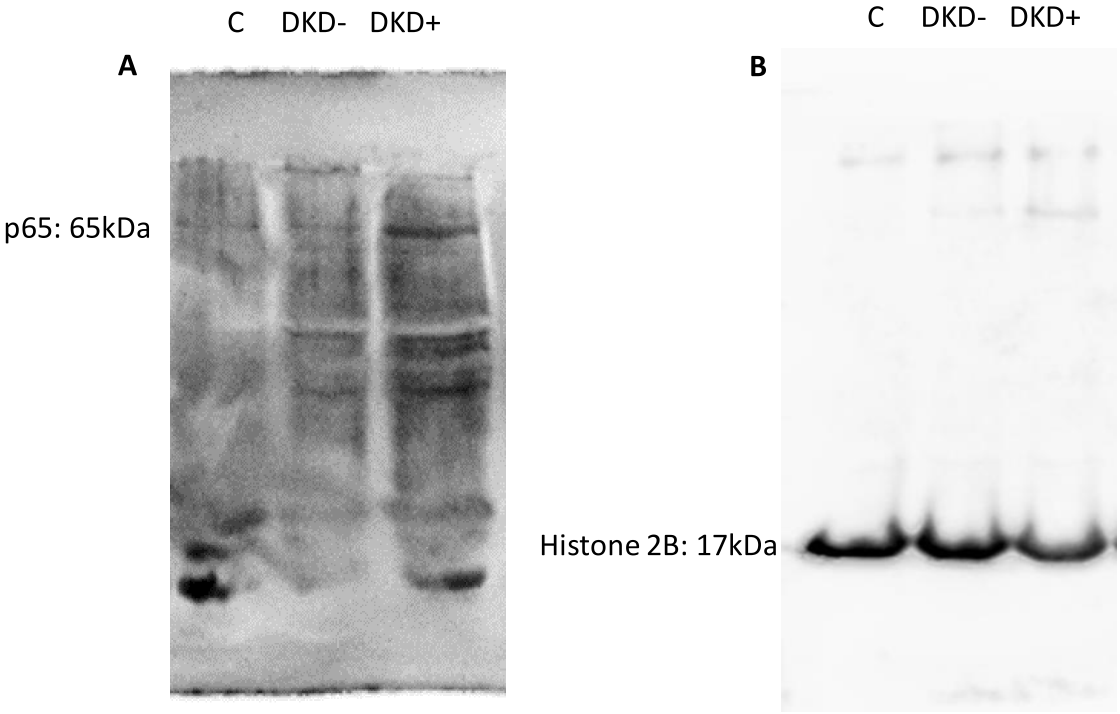

Full-length phosphorylated p65 (**A**) and constitutive Histone 2B (**B**) representative Western Blots of groups C, DKD- and DKD+.

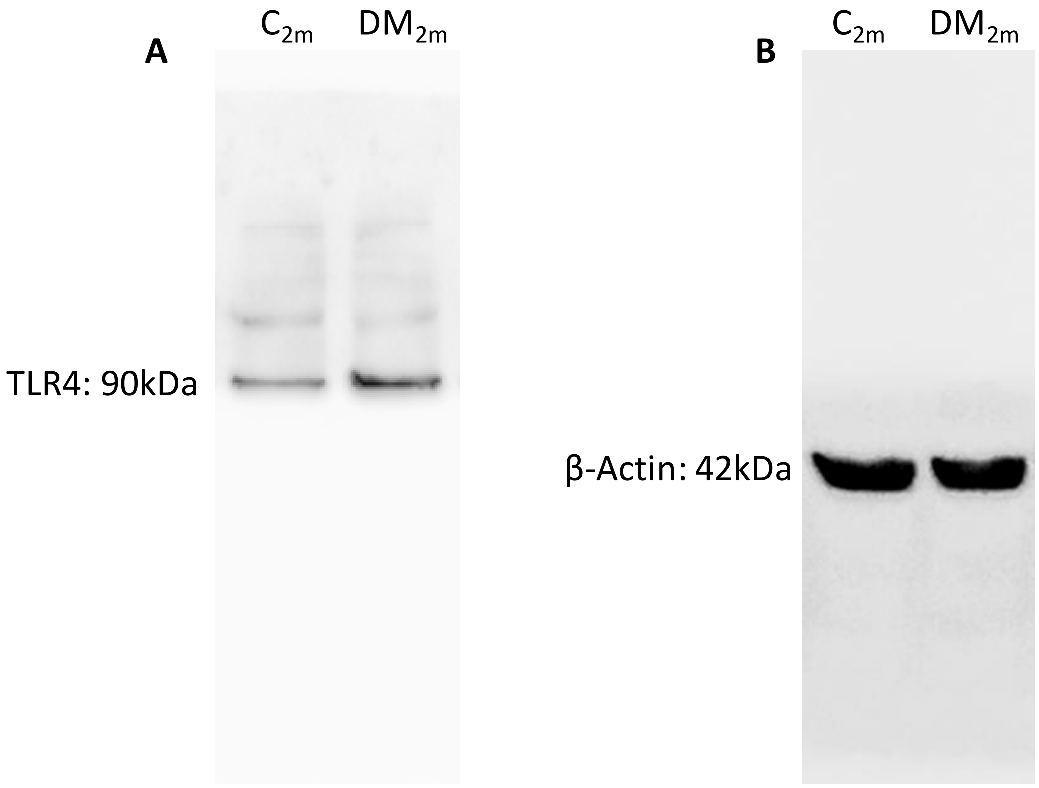

Full-length TLR4 (**A**) and constitutive β-Actin (**B**) representative Western Blots of groups C_{2m} and DM_{2m}.

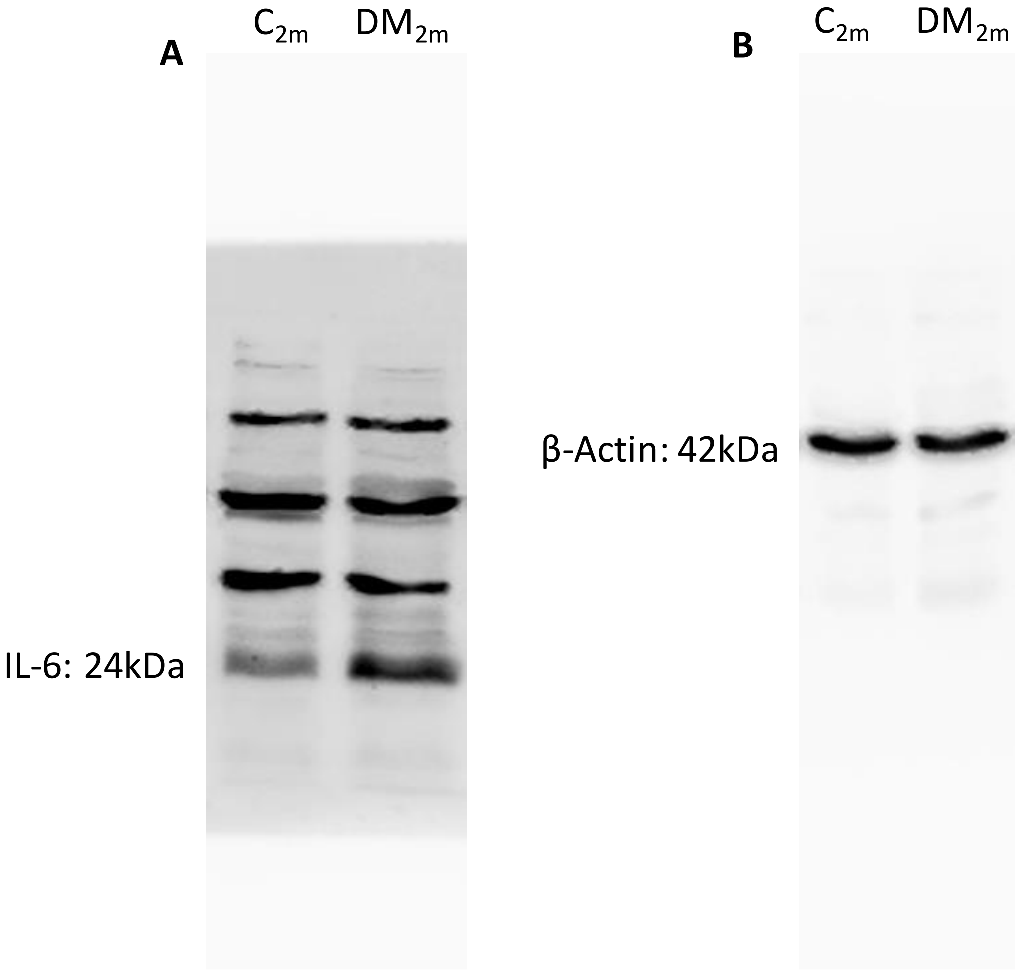

Full-length IL-6 (**A**) and constitutive β-Actin (**B**) representative Western Blots of groups C_{2m} and DM_{2m}.

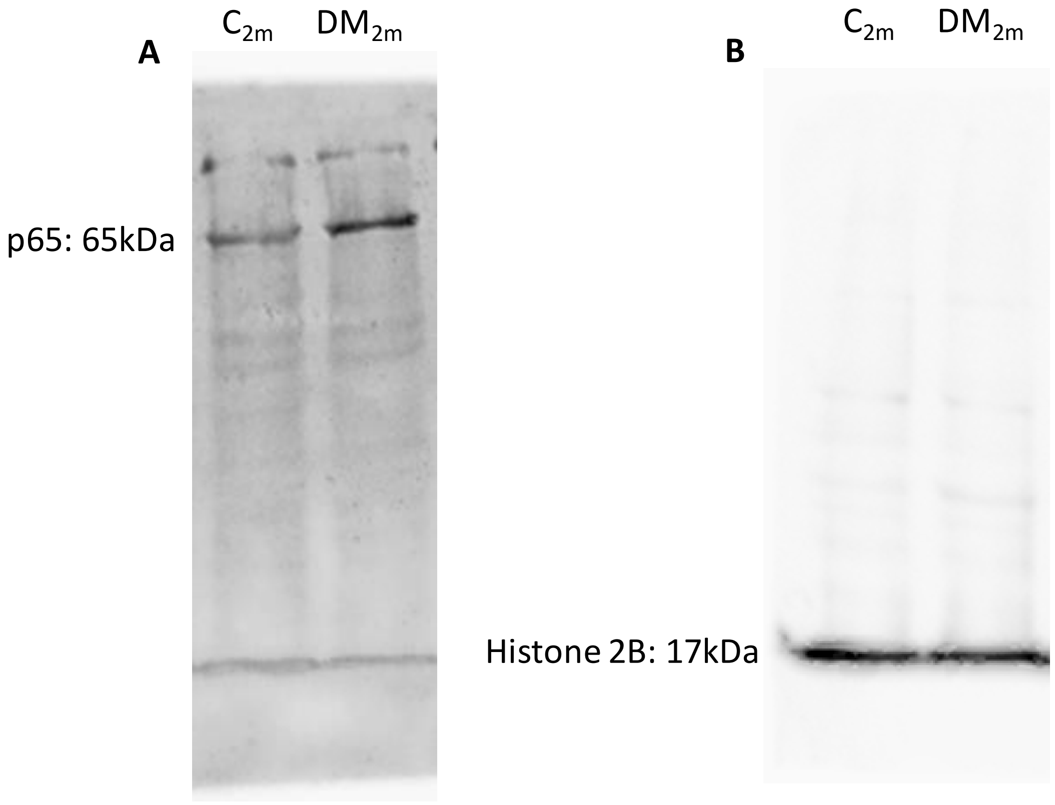

Full-length phosphorylated p65 (**A**) and constitutive Histone 2B (**B**) representative Western Blots of groups C_{2m} and DM_{2m}.

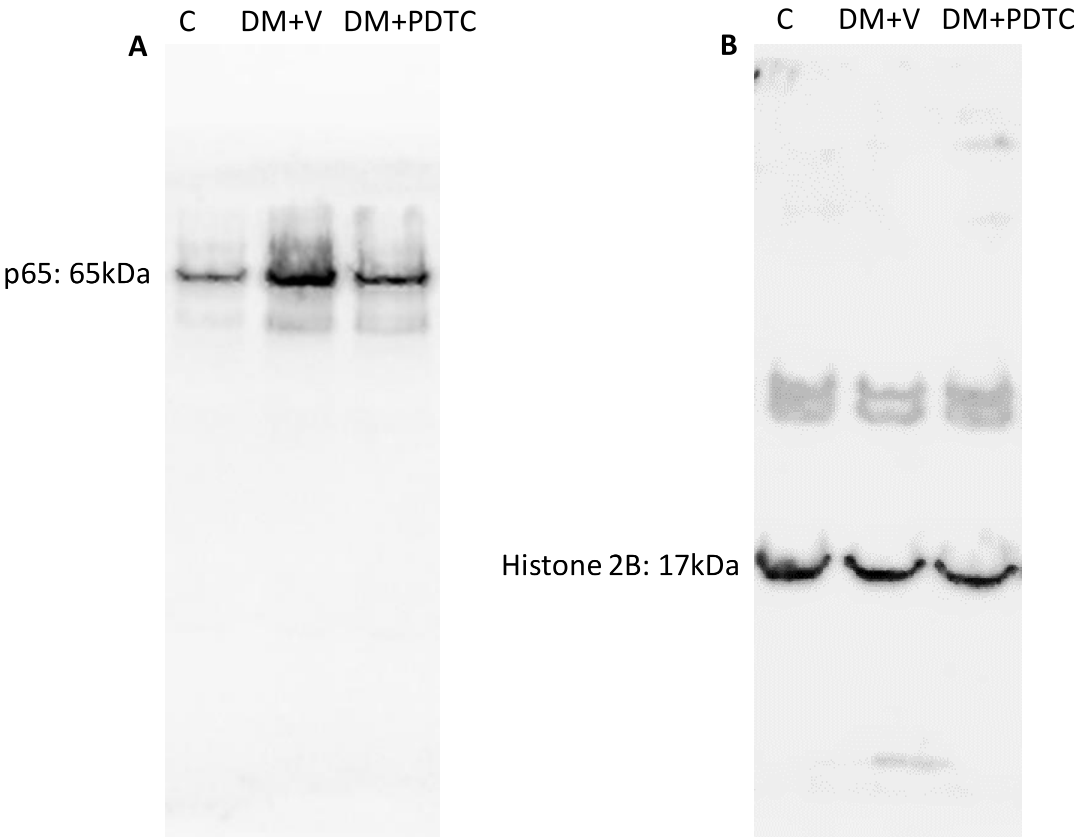

Full-length phosphorylated p65 (**A**) and constitutive Histone 2B (**B**) representative Western Blots of groups C, DM+V and DM+PDTC.

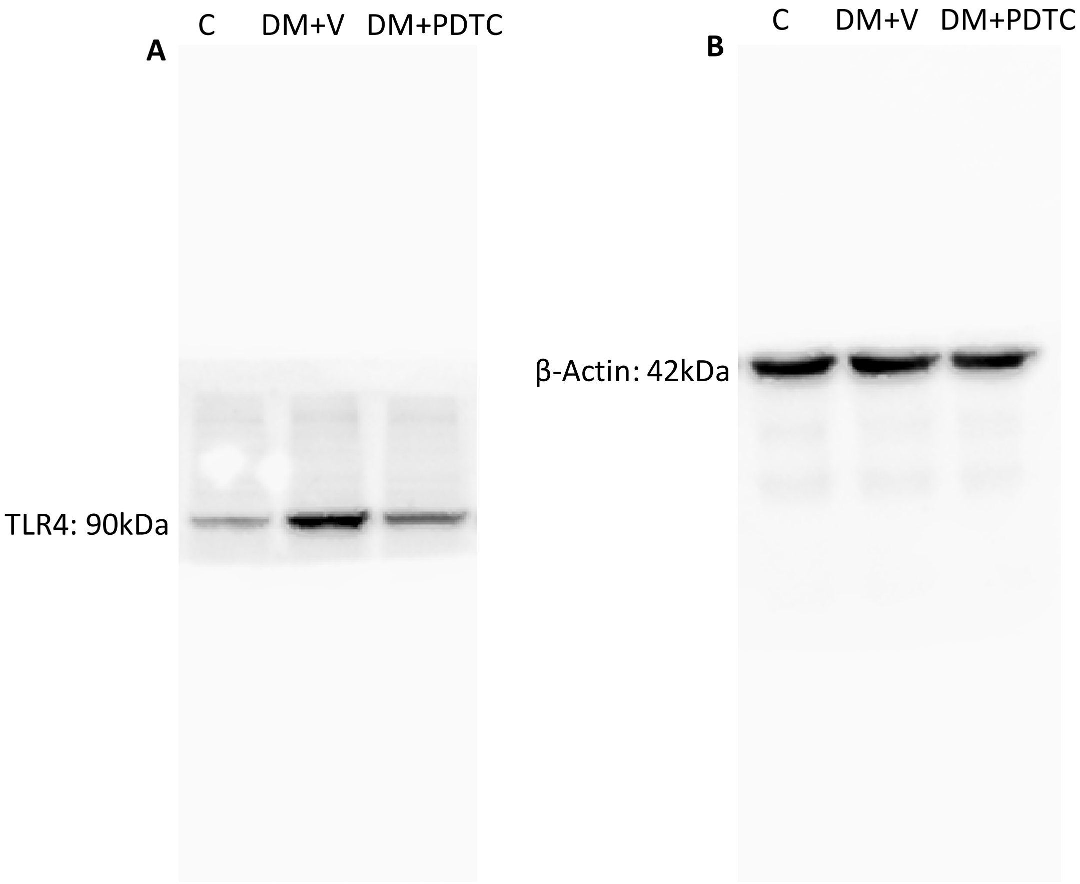

Full-length TLR4 (**A**) and constitutive β-Actin (**B**) representative Western Blots of groups C, DM+V and DM+PDTC.

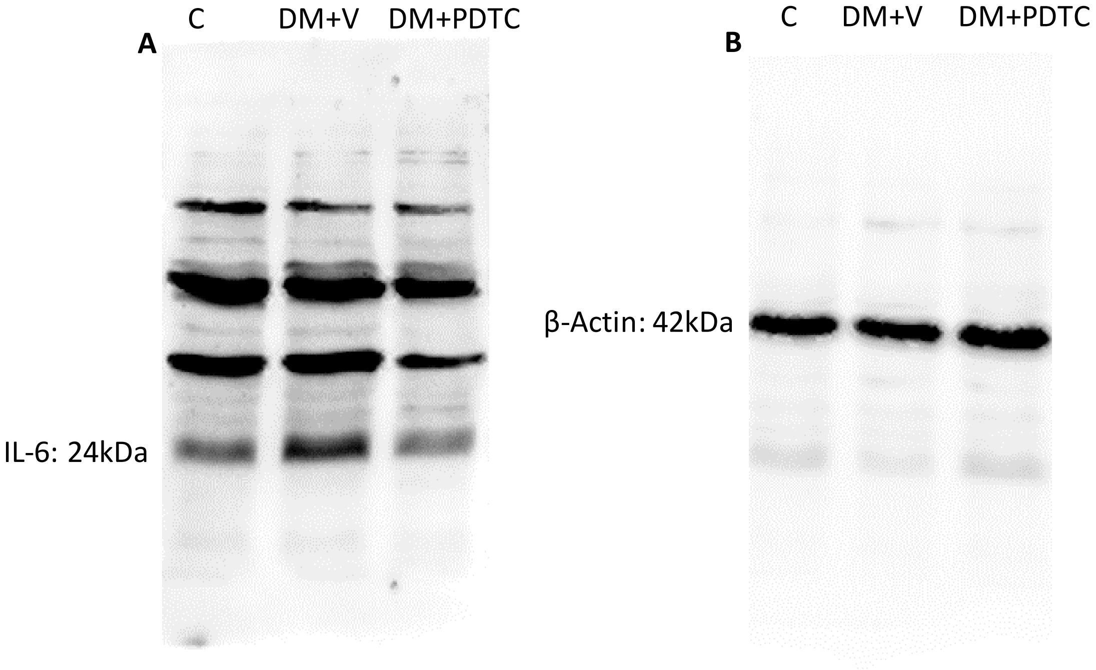

Full-length IL-6 (**A**) and constitutive β-Actin (**B**) representative Western Blots of groups C, DM+V and DM+PDTC.

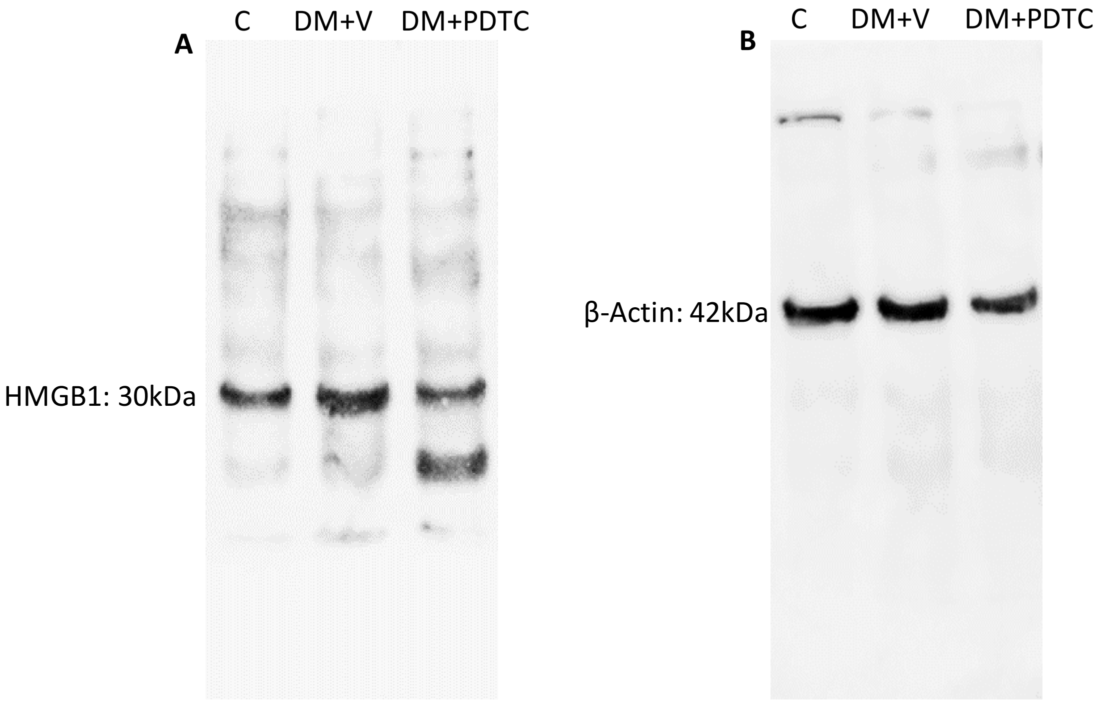

Full-length HMGB1 (**A**) and constitutive β-Actin (**B**) representative Western Blots of groups C, DM+V and DM+PDTC.

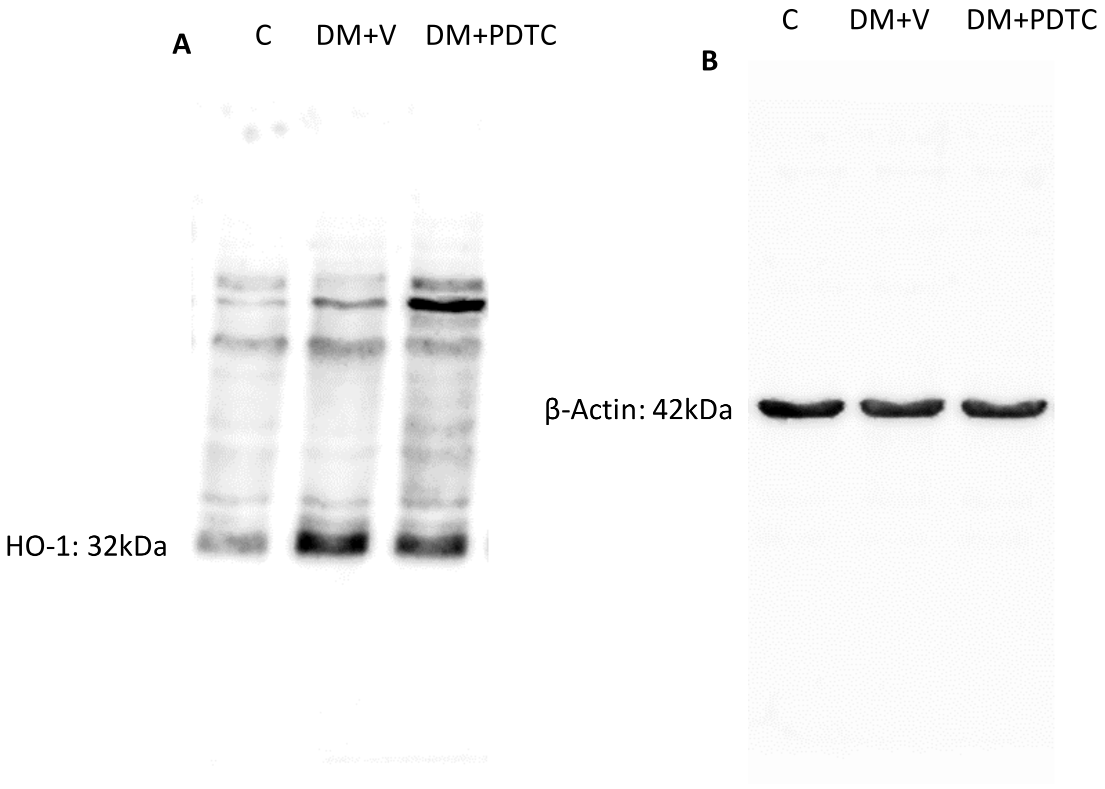

Full-length Heme Oxigenase-1 (HO-1) (**A**) and constitutive β-Actin (**B**) representative Western Blots of groups C, DM+V and DM+PDTC.

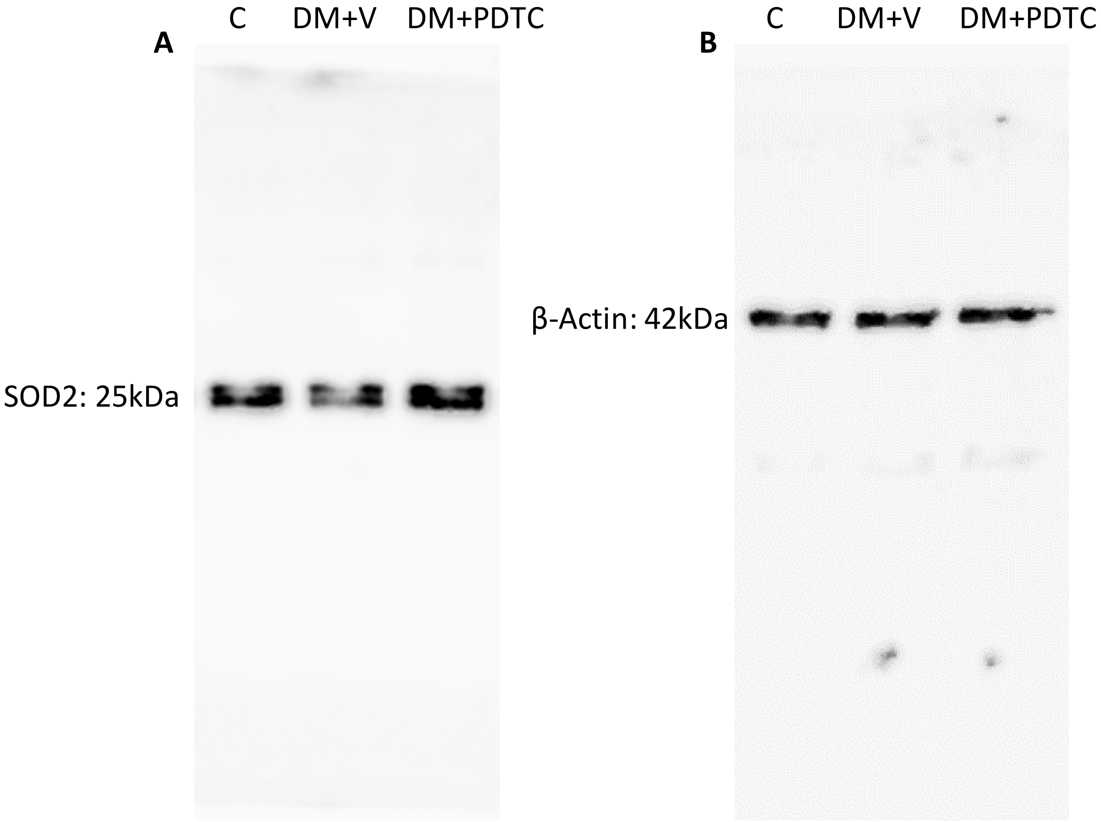

Full-length Superoxide Dismutase 2 (SOD2) (**A**) and constitutive β-Actin (**B**) representative Western Blots of groups C, DM+V and DM+PDTC.
