# Supplementary material for: NF-κB System Is Chronically Activated and Promotes Glomerular Injury in Experimental Type 1 Diabetic Kidney Disease
Source: Front Physiol. 2020 Feb 11;11:84. doi: 10.3389/fphys.2020.00084 (PMC7026681; doi:10.3389/fphys.2020.00084)
Supplement: Supplementary file 2 [file Data_Sheet_1.PDF]

Supplementary Figures

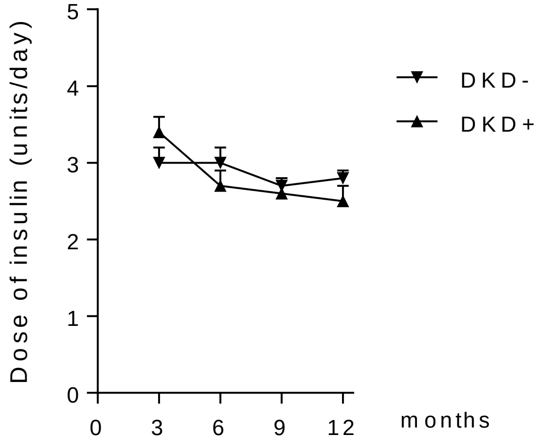

Supplementary Figure 1. Insulin doses (units/day) needed to maintain blood glucose levels between 350 and 450 mg/dL in groups DKD- and DKD+. No difference was observed between groups DKD- and DKD+ along the study.

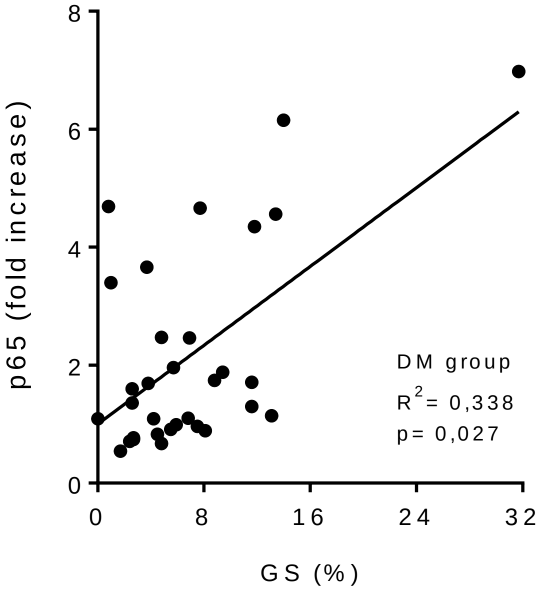

Supplementary Figure 2. Correlation (Pearson's coefficient) between the nuclear expression of p65 (fold increase) and the percentage of sclerotic glomeruli (GS, %) in the DM group (n=32).
